# Reassessment of sustainable rural tourism strategies after COVID-19

**DOI:** 10.3389/fpsyg.2022.944412

**Published:** 2022-07-29

**Authors:** Fatma Kürüm Varolgüneş, Faysal Çelik, María de la Cruz Del Río-Rama, José Álvarez-García

**Affiliations:** ^1^Department of Architecture, Bingöl University, Bingöl, Turkey; ^2^Department of Urban Studies, Gaziantep University, Gaziantep, Turkey; ^3^Departmentof Business Management and Marketing, Faculty of Business Sciences and Tourism, University of Vigo, Ourense, Spain; ^4^Departamento de Economía Financiera y Contabilidad, Instituto Universitario de Investigación para el Desarrollo Territorial Sostenible (INTERRA), Universidad de Extremadura, Cáceres, Spain

**Keywords:** sustainable rural tourism, COVID-19 pandemic, decision-making, analytic hierarchy process (AHP), A’WOT, TOWS, Gökçeada/Turkey

## Abstract

This study aimed to develop indicators that measure rural tourism destinations in a sustainable framework during the COVID-19 process. In order to achieve this goal, the A’WOT and TOWS hybrid method was used in the study. In line with this goal, the priority order was calculated by determining the factors for strengths, weaknesses, threats and opportunities. Once these factors have been identified, strategies have been developed to build on strengths and eliminate weaknesses, while taking advantage of the opportunities and countering threats. In the study, Gökçeada-Turkey, which has recently come to the fore with its rural tourism potential, has been considered as a destination area, and strategies have been developed that adopt sustainable and responsible tourism approaches and increase the roles and capabilities of local communities. The results obtained in the study are expected to be meaningful for other rural destinations that are similar to Gökçeada.

## Introduction

The tourism sector has been one of the sectors most affected by the COVID-19 outbreak due to economic uncertainty and travel restrictions (Higgins-Desbiolles, 2020; Jamal and Budke, 2020; Yang et al., 2020). UNWTO (2021) reported that in February 2021, there was a 90% decrease in international tourist mobility. Besides, UNWTO (2021) stated that there were 1.4 billion tourists worldwide in 2019, and further announced that the main destinations (i.e., France, Spain, and the United States) were the countries most affected by the pandemic in terms of the spread of the epidemic and economic damage (UNWTO, 2021). Despite these negativities, some researchers consider the post pandemic recession an opportunity to re-start the tourism industry, and develop more sustainable strategies by eliminating the negative consequences such as economic exploitation and overcrowding (Brouder, 2020; Niewiadomski, 2020; Farmaki, 2021; Vărzaru et al., 2021).

Similarly, some researchers have suggested that more localized forms of tourism will be the preferred alternative in the future (Higgins-Desbiolles, 2020). The increasing interest in accommodations and alternative tourism services in rural destinations for local tourists, along with the negative impacts of mass tourism in the 2020 summer season, confirms this idea. Visitors’ preference toward destinations that have a lower density of tourist population, that are far from big cities, is considered an opportunity for the development of the economy for some rural areas. Even after the relaxation of movement restrictions in many European countries, there was a limited recovery in domestic tourism (Marques et al., 2021). In particular, activities such as second housing, cycling, hiking, nature visits, water sports, camping, etc., are the types of tourism people prefer (Seraphin and Dosquet, 2020). With these developments, the damage caused by mass tourism, which has been on the agenda for a long time, and calls to turn to alternative tourism types have come to the fore again during the epidemic process. Although the concept of sustainability appears in many different social dimensions, the common feature of all these concepts is that they focus on the future of people and aim to protect the resources of the areas considered as the living space of people. Sustainable tourism involves social responsibility, economic efficiency, and environmental sensitivity at every stage. In this context, various definitions such as soft tourism, ecological tourism, nature tourism and rural tourism are used (Beyhan and Ünügür, 2010). These types of tourism are developing in a direction of change that preserves ecological balances, protects future generations, brings social values to the fore and increases regional income. In this study, the focus is on rural tourism, which is one of the sustainable tourism types. Bringing examples of rural tourism from various parts of the world to the literature with their natural environment, architecture and cultural structure will make a significant contribution to the development and sustainability of rural tourism. Since tourism is an important tool for economic growth and diversification, the development of alternative tourism types, especially in rural areas, is important for national economies in terms of income and employment opportunities (Sharpley, 2002; Mugauina et al., 2020). Recently, an increasing number of scientists have been interested in rural heritage and communities (Gullino and Larcher, 2013; Zou et al., 2014; Gao and Wu, 2017). Some researchers have suggested that rural tourism destinations develop uncontrolled and they emphasize that this uncontrolled growth will cause environmental, social and economic problems (Cánoves et al., 2004). Therefore, it is important to focus on sustainability in tourism. Reasons such as the deterioration of the ecological balance due to global warming, the loss of social values, and the inability to protect natural, historical, social and cultural assets make sustainable tourism a necessity (Kişi, 2019; Filipiak et al., 2020).

In this context, the aim of the study is to raise awareness for the kind of development that will not damage the local architecture and texture, nor harm the nature in rural destinations mostly preferred by local tourists during the COVID-19 process. In order to achieve this goal, A’WOT hybrid methods were used in the study. In line with this goal, the strengths and weaknesses of bringing local architecture to tourism have been revealed, and the priority order has been calculated by determining the threats and opportunities that may be encountered. In the study, Gökçeada-Turkey, which has recently come to the fore with its rural tourism potential, has been considered as a destination area and strategies have been developed that adopt sustainable and responsible tourism approaches and increase the roles and capabilities of local communities. It is expected that the results obtained in the study will be an example for other rural destinations that are similar to Gökçeada.

## Literature review

### Sustainable rural tourism

In recent years, activities related to rural tourism have increased in many countries and rural tourism types have become an alternative to mass tourism (Busby and Rendle, 2000). Vaishar and Št’astná (2020) point out that the disaster scenarios in tourism after the COVID-19 pandemic are mainly related to urban destinations that focus on foreign tourism, and draw attention to the increase in rural destinations with their study in Czechia (Vaishar and Št’astná, 2020). In their study, Zhu and Deng (2020) and Li et al. (2021) test various hypotheses that the tendency to rural tourism has increased in China, considering both cost and safety in the context of the globally life-threatening COVID-19 pandemic (Zhu and Deng, 2020; Li et al., 2021). In their study, Wen et al. (2020) investigated the impact of the development of new tourism markets such as health tourism, slow tourism and smart tourism on the consumption patterns of Chinese tourists with the impact of COVID-19 (Wen et al., 2020). Likewise, Higgins-Desbiolles (2020), argues that COVID-19 offers an opportunity to shift the tourism paradigm toward sustainability and local interests. Moreover, they suggest that in order to build the future, special attention should be paid to increasing resilience and promoting sustainability at all levels. These and similar studies have increased the interest in rural tourism. Rural tourism has an innovative and sustainable approach that preserves the local texture and identity while targeting rural development (Akgün et al., 2015; Št’astná et al., 2020).

Rural tourism takes an innovative and sustainable approach that preserves local structure and identity while targeting rural development and it is a form of tourism based on natural resources and intertwined with rural settlements. Due to its many positive effects, its importance in tourism is better understood day by day. Different climates, natural environments and different cultures all over the world guide this type of tourism. For this reason, it is seen that there are different approaches to the definition of rural tourism in the literature and there is no consensus on a common definition (Sharpley and Roberts, 2004; Aref and Gill, 2009; Carneiro et al., 2015; Lane and Kastenholz, 2015; Wegren, 2016; Ayhan et al., 2020). Although there are different definitions of rural tourism, they all have in common that it plays a major role in protecting and promoting the world’s natural and cultural heritage. Thus, rural tourism is a form of tourism compatible with sustainable tourism. Rural tourism jointly evaluates rural areas’ economic, social, and environmental components. It is closely linked to people, places, and products. It has unique impacts on the environment and economic growth (Yang et al., 2021). The development of tourism activities can have positive impacts such as creating jobs, improving the quality of life for local people, enhancing the public image of the region, preserving cultural heritage, and even developing business networks. However, negative impacts such as ecological damage, depletion of local resources, and infrastructure congestion must also be considered (Yang et al., 2021).

In her study, Topçu (2018) aimed to find the most appropriate planning strategy for the sustainability of Birgi’s local character and identity by taking advantage of Birgi’s strong natural environment and cultural identity (Topçu, 2018). She used A’WOT analysis, a combination of the Analytical Hierarchy Process (AHP) and SWOT analysis, to identify these strategies. Then, with the TOWS matrix, he presented suggestions for achieving a sustainable tourism industry in Birgi and preserving the local identity. Likewise, Kişi (2019) put forward strategies for the development of sustainable tourism in touristic destinations and used A’WOT analysis to emphasize the priority order of these strategies. In her work, she emphasized sustainability criteria such as minimizing negative environmental and social impacts, reducing carbon footprint, normalizing the behavior of visitors, reducing tourist overcrowding, compensating for negative effects caused by tourism, and considering the needs of local people (Kişi, 2019). Sulistyadi et al. (2017), used SWOT analysis and a quantitative strategic planning matrix to create a sustainable tourism development model with his case study at the Thousand Islands Tourism Zone in Jakarta (Sulistyadi et al., 2017). In examining the current studies, potentials, evaluations, expectations and strategies for rural tourism developed in different regions using various quantitative methods are considered. Tourism studies in this range show that he A’WOT analysis approach can be applied actively in order to determine the priorities of qualitative alternatives that are difficult to translate simply into quantitative figures, and that the approach can ultimately lead to more systematic and feasible showing decisions (Lee et al., 2021).

Rural areas with cultural, historical, artistic and architectural background are disappearing or being assimilated, especially in developing countries (Gao and Wu, 2017). This should be taken into account when determining new destinations. In developing rural destinations for tourism, an approach should be taken that preserves the ecological balance, protects future generations, prioritizes social values, and increases regional income. Unless this point of view is maintained, any new potential mass discovered will continue to be the victim of tourism exploitation. For this reason, the types of tourism proposed in rural areas should be developed following the shared knowledge and opinions of all stakeholders (professionals, locals, local governments, scientists, etc.). In this awareness, Gökçeada, whose natural and cultural values have not been discovered yet, was preferred as a research area. With the introduction of the local identity values of Gökçeada at the global scale, attention was drawn to its protection as a cultural heritage. In the study, the SWOT factors were determined based on expert opinions. Then, these factors were prioritized with the AHP method. Finally, the strategies for developing sustainable rural tourism are presented using the TOWS matrix in line with the region-specific vision and key sustainable tourism objectives (Kişi, 2019).

### The relationship between rural architecture and rural tourism in Gökçeada (Turkey)

Rural architecture can be defined as architecture produced based on traditions, using natural environmental features, social and cultural structure, local materials, and local construction techniques (Singh et al., 2009). The local residence, which means the meeting of the rural culture bearing the traces of the past with architecture, exists with its own unique identities as it reflects the culture, social relations and habits of ordinary people in their daily life, and the ordinary tastes, beliefs and life priorities of the owner and master. Rural settlements attract attention with their preserved natural environments, architectural identities and original structures (Anna-Maria, 2009). These settlements are preferred to get away from the city, to rest, to visit, and to see. Gökçeada, which has the characteristics of Anatolian-Greek settlement, has preserved its culture and rural texture and brought it to the present day. In this context, Gökçeada has become one of the important points of Western Anatolia in terms of rural and cultural tourism for alternative holiday enthusiasts different from mass tourism (Karayel, 2019). On the other hand, its nature, history, calmness, architecture, festivals, sports activities and proximity to touristic canters further increase the touristic importance of Gökçeada. Stone houses, cobblestone streets, churches, laundries, squares where coffee and various shops are gathered form the architectural texture of the villages (Canan and Kürüm Varolgüneş, 2017).

**FIGURE 1:**
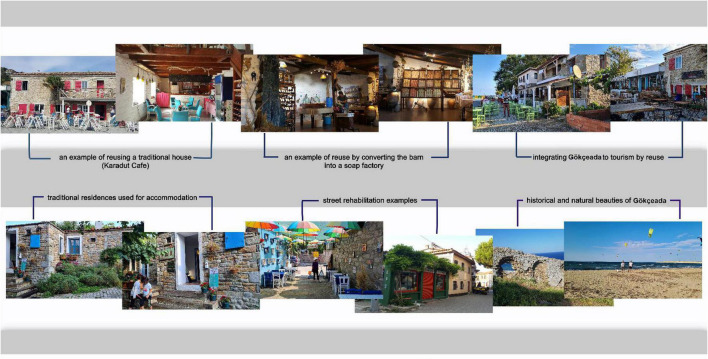
Examples of traditional architecture that add value to tourism from Gökçeada (photographs by Kürüm Varolgüneş, 2020).

The restoration of traditional houses and their reuse by converting them into lodging establishments, entertainment facilities, and commercial spaces is an important approach to protecting these buildings (Dündar, 2012). It is seen that tourists who come to the region usually prefer these houses, which characterize the fabric of the island, as accommodation. Rural architecture, an accelerating factor in tourism development, has thus acquired another important role. As a result of the rapid increase in its touristic potential in recent years, olive growing, viticulture and wine making, which are the livelihoods of Gökçeada, have become important business lines that contribute to tourism (Çalışkan, 2010). The annual festivals during the grape harvest attract many local and foreign tourists to this region. Olive cultivation has also gained touristic importance. Olive oil, natural soaps, ceramics and porcelain related to olives are marketed as touristic products. In summary, the rural architectural heritage has enabled rural tourism development in Gökçeada. In parallel with the developing tourism phenomenon, all the settlement resources are used for tourism (Figure 1).

## Materials and methods

The A’WOT method, first proposed by Kurttila et al. (2000), is a hybrid method that combines AHP and SWOT analysis. By incorporating the AHP technique into SWOT analyses, SWOT groups and factors are made measurable and their priorities are presented numerically (Kurttila et al., 2000; Akbulak and Cengiz, 2014). This is achieved by the pairwise comparisons of SWOT factors in the AHP technique and the eigenvalue calculations approach (Kurt, 2020). Thus, it becomes possible to consider a new alternative strategy that expresses an existing or expected situation in more detail. In the A’WOT technique, SWOT analyses are carried out in the first stage (Kajanus et al., 2004). For this, first of all, SWOT groups consisting of strengths, weaknesses, opportunities and threats are formed. The factors belonging to each SWOT group are then ranked as objectively as possible. The factors of the internal and external environment thus obtained are included in the SWOT analysis. Then, pairwise comparisons are made between the factors in each SWOT group. According to the information obtained from these comparisons, the relative importance (priorities) of the factors is calculated using the eigenvalue approach within the scope of the AHP technique. Pairwise comparisons are then performed between the four SWOT groups (Kurttila et al., 2000; Kajanus et al., 2004). This is done separately for each of the four SWOT groups. As a result, the overall priority values of all SWOT factors, whose total value is equal to one, are obtained (Güngör, 2018; Lee et al., 2021). The A’WOT method is applied with a systematic approach. Pairwise comparisons of the determined SWOT criteria are performed. This comparison is based on Saaty’s 9-point scale for analytical efficiency (Saaty, 1987; Table 1). Pairwise comparisons of the generated criteria are arranged into an n × n square matrix. The diagonal elements of the matrix are equal to “1” (Wu et al., 2010). If the value of the element (i, j) is greater than 1, the criterion in row (i) is better than the criterion in column (j); otherwise, the criterion in column (j) is better than in row (i). The (j, i) element of the matrix is the inverse of (i, j). The base eigenvalue and corresponding normalized right eigenvector of the comparison matrix give the relative importance of the various criteria being compared. The elements of the normalized eigenvector are called “weights” according to criteria or sub-criteria, and “ratings” according to alternatives (Bhushan and Rai, 2004; Bafail and Abdulaal, 2021).

**TABLE 1:** Saaty’s 1–9 scale for pairwise comparison.

Weight intensity	Definition	Explanation
1	Equally important	Two activities contribute equally to the objective
3	Moderately important	Experience and judgment slightly favor one over another
5	Strongly important	Experience and judgment strongly favor one over another
7	Very Strongly important	An activity is strongly favored and its dominance is demonstrated in practice
9	Extremely important	The importance of one over another affirmed on the highest possible order
2,4,6,8	Intermediate weights	Used to represent compromise between the priorities listed above

The consistency of the matrix of order “n” is evaluated. Comparisons made by this method are subjective and the AHP tolerates inconsistency through the amount of redundancy in the approach. If this consistency index fails to reach a required level, then the answers to comparisons may be re-examined (Thungngern et al., 2017). Where λ max is the maximum eigenvalue of the judgment matrix. This CI can be compared with that of a random matrix RI. The RI values are fixed numbers and determined by “*n*” values. Then, the ratio derived CI/RI is termed the consistency ratio (CR) (Kurt, 2020; Eryürük et al., 2021).


(1) $$\text{Consistency}\,\text{Index}\,\left(\text{CI}\right)=\frac{\lambda_{m\,a\,x}-n}{n-1}$$

(2) $$\text{Consistency}\,\text{Ratio}\,\left(C\,R\right)=\frac{\mathrm{Consistency}\,\mathrm{Index}\,\left(\mathrm{CI}\right)}{\mathrm{Random}\,\mathrm{consistency}\,\mathrm{Index}\,\left(\mathrm{RI}\right)}$$

**Table:** 

*n*	1	2	3	4	5	6	7	8	9	10
RI	0	0	0.58	0.9	1.12	1.24	1.32	1.41	1.45	1.49

**FIGURE 2:**
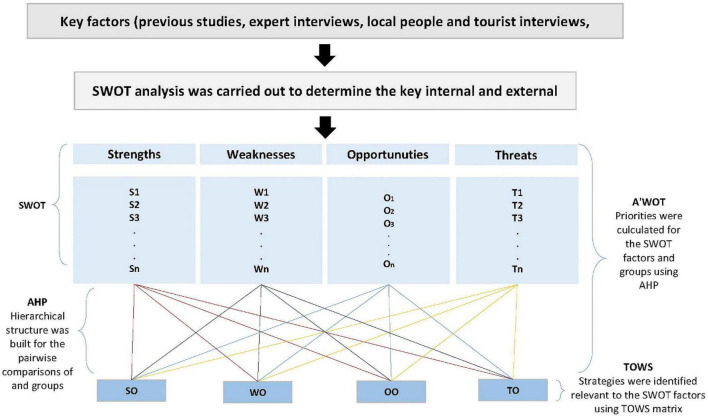
The structure of the applied method (edit by authors from Asadpourian et al., 2020).

Random Consistency Index (RI);

The result is considered reliable if the CR value is usually less than “0.1.” Otherwise, minimization of errors is accomplished by repeating a pairwise comparison (Saaty, 1987, 1990; Razavi et al., 2011; Kamaruzzaman et al., 2018). This process is repeated until sufficient consistency is achieved. Some researchers have also benefited from the TOWS matrix together with A’WOT while determining strategies for tourism (Akbulak and Cengiz, 2014; Topçu, 2018; Kişi, 2019; Asadpourian et al., 2020; Özgeriş and Karahan, 2021). The TOWS matrix is a quantitative strategic planning matrix. The TOWS matrix (Weihrich, 1982) is formulated according to the SWOT factors with the highest priority values from each SWOT group. By using the priority of strengths with the TOWS matrix, strategies for eliminating weaknesses, obtaining the opportunity and eliminating the threat are presented. In this study, the A’WOT method, which is used to determine sustainable rural tourism goals, is strengthened with the TOWS matrix. TOWS analysis makes it possible to develop strategies on how to take advantage of positive situations to overcome the negative aspects of the current situation (Topçu, 2018). The structure of the applied method is summarized in Figure 2.

## Results and discussion

There has been a substantial increase in interest in rural tourism after COVID-19. In the study, strategies for the development of sustainable tourism in Gökçeada, which was caught unprepared for this increase with various analyses, were presented. First of all, previous studies were reviewed, interviews were held with academicians, experts in institutions and local people, and SWOT factors were determined by taking personal professional experiences into account. Eight sub-factors for strengths, six sub-factors for opportunities, and seven sub-factors for weaknesses and threats were determined in the SWOT analysis and are presented in Table 2.

**TABLE 2:** SWOT factors of the Gökçeada in terms of sustainable rural tourism.

SWOT groups		SWOT factors
Strengths (W)	S1	The effectiveness of traditional architecture
	S2	Natural beauty (Desire to be within natural life.)
	S3	The fact that the social and cultural identity of Gökçeada has not yet deteriorated
	S4	The friendliness and hospitality of the island people
	S5	The continuation of intercultural interaction on the island
	S6	Popular destination
	S7	Applicability of rural tourism for 12 months
	S8	Fertile soils that offer a variety of products
Weaknesses (W)	W1	Lack of knowledge and entrepreneurship of the locals
	W2	Lack of promotion and marketing activities in the region
	W3	Insufficient number of accommodation facilities
	W4	Inefficient use of existing agricultural potential
	W5	The fact that the carrying capacity for tourism on the island has not been determined
	W6	Lack of coordination and communication among stakeholders. (local people, non-governmental organizations, local government and public)
	W7	Lack of qualified workforce
Opportunities (O)	O1	Increasing the interest of local tourists to the region
	O2	High agricultural productivity
	O3	Having renewable energy sources
	O4	Potential to host festivals, summer schools, workshops and various events
	O5	Poor transport links that preserve the island fabric
	O6	Creating accommodation opportunities suitable for the natural and cultural texture of the region
Treats (T)	T1	Natural hazards such as earthquakes, floods, landslides
	T2	The tourism concept being more identified with the coastal tourism in Turkey
	T3	Losing the original cultural and social values of the villages
	T4	Political and economic instability on the island
	T5	The disappearance of unprotected examples of civil architecture on the island
	T6	Young population leaving the island
	T7	Insufficient investment for the island

Once these factors have been identified, strategies have been developed that can build on strengths, eliminate weaknesses, take advantage of opportunities, and counter threats (Kurt, 2020). Strengths are features that add value to the area. They provide the environmental, social and cultural values of the island with a positive potential for rural tourism. Weaknesses are internal factors that can have negative effects for the region. The lack of information and market, the fact that the current potential has not been evaluated and supported until now have been accepted as the difficulties in front of the development of tourism. Opportunities, the island being untouched, preserving its natural texture and having cultural heritage are external positive factors for the development of rural tourism. Threats are external obstacles that are largely out of control. The SWOT analysis made provides an overview. The factors determined by the SWOT analysis form the basis for the quantitative techniques that are considered to develop sustainable strategies in rural tourism. AHP, which is a pairwise comparison method, was used to determine the importance of the resulting SWOT factors. In this section, which constitutes the second stage of the study, the importance levels of all sub-factors were calculated separately. For evaluations, an AHP comparison survey was conducted with 10 tourism experts, 7 academics, 3 local managers and 5 business owners in July and August 2020, when COVID-19 was effective. As a result of the evaluations made by the experts, the arithmetic mean of the results was taken to reach a common result. Table 2 lists the results of the analysis of the relative importance among the SWOT factors and the consistency analyses for the development of sustainable strategies for rural tourism in Gökçeada.

The pairwise comparison matrix shows the importance levels of the criteria in comparison with each other within a certain logic. The criteria are converted into a matrix through pairwise comparisons (Eryürük et al., 2021). Then, the synthesis of the pairwise comparison matrices is done. In the synthesis process, each element of the matrix is divided by the sum of the column it belongs to and the normalized matrix is formed. The priority vector is obtained by taking the arithmetic average of each row of the normalized matrix. No matter how consistent the AHP is within itself, the realism of the results will naturally depend on the consistency of the one-to-one comparison between the criteria by the decision maker. AHP proposes a process for measuring consistency in these comparisons. With the Consistency Ratio obtained as a result, it provides the opportunity to test the consistency of the priority vector found and therefore the one-to-one comparisons (decisions) made between the criteria. AHP bases its calculation on the comparison of the number of criteria and a coefficient called the eigenvalue (λ). In order to calculate (λ), firstly the comparison matrix (A) and the priority vector (W) are multiplied. In the examination of the consistency ratios of the SWOT groups, it was determined that the decision matrix among the SWOT main factors was consistent with 0.0367 (CR), in pairwise comparisons of the SWOT sub-factors, the decision matrix was consistent with 0.0935 (CR) for strengths, 0.0057 (CR) for weaknesses, 0.0953 (CR) for opportunities, and 0.0969 (CR) for threats. The most important priority was calculated as “Strengths” with a weight value of 0.5205, followed by “Opportunities” 0.2971, “Weaknesses” 0.1244, and “Threats” 0.058. The results of the analysis are presented in Tables 3, 4. When the priorities of the factors belonging to the SWOT groups are examined, the strongest factor in the development of rural tourism is “Natural beauty (Desire to be within natural life) (S2)” with a weight of 0.2987, followed by “The effectiveness of traditional architecture (S1)” with a weight of 0.1767, “Popular destination (S6)” with a weight of 0.123 and a weight of 0.1093 “Continuation of intercultural interaction on the island (S5)” are listed as strong factors.

**TABLE 3:** AHP factors and descriptions in SWOT-Matrix (weight of SWOT factors).

	S	W	O	T	λmax		S	W	O	T	Row averages
S	1.00000	5.00000	2.00000	7.00000	2.1431	S	0.5426	0.5357	0.5660	0.4375	0.5205
W	0.20000	1.00000	0.33333	3.00000	0.5017	W	0.1085	0.1071	0.0943	0.1875	0.1244
O	0.50000	3.00000	1.00000	5.00000	1.2209	O	0.2713	0.3214	0.2830	0.3125	0.2971
T	0.14286	0.33333	0.20000	1.00000	0.2333	T	0.0775	0.0357	0.0566	0.0625	0.0581
Total	1.84286	9.33333	3.53333	16.00000	4.0990	Total	1.0000	1.0000	1.0000	1.0000	1.0000

CR = 0.0367 < 0.1.											

**TABLE 4:** Weight of SWOT sub factors.

	S1	S2	S3	S4	S5	S6	S7	S8	λmax	S1	S2	S3	S4	S5	S6	S7	S8	Row averages
S1	1.0000	0.2000	3.0000	2.0000	1.0000	4.0000	3.0000	2.0000	1.56742	0.11215	0.05172	0.26087	0.16000	0.12245	0.35294	0.18750	0.13333	0.17669
S2	5.0000	1.0000	3.0000	1.0000	3.0000	3.0000	3.0000	3.0000	2.57968	0.56075	0.25862	0.26087	0.08000	0.36735	0.26471	0.18750	0.20000	0.29872
S3	0.3333	0.3333	1.0000	2.0000	1.0000	0.5000	2.0000	2.0000	0.82410	0.03738	0.08621	0.08696	0.16000	0.12245	0.04412	0.12500	0.13333	0.08952
S4	0.5000	1.0000	0.5000	1.0000	0.5000	0.3333	1.0000	2.0000	0.78927	0.05607	0.25862	0.04348	0.08000	0.06122	0.02941	0.06250	0.13333	0.08813
S5	1.0000	0.3333	1.0000	2.0000	1.0000	1.0000	3.0000	3.0000	1.11793	0.11215	0.08621	0.08696	0.16000	0.12245	0.08824	0.18750	0.20000	0.10933
S6	0.2500	0.3333	2.0000	3.0000	1.0000	1.0000	2.0000	1.0000	0.98946	0.02804	0.08621	0.17391	0.24000	0.12245	0.08824	0.12500	0.06667	0.12314
S7	0.3333	0.3333	0.5000	1.0000	0.3333	0.5000	1.0000	1.0000	0.50385	0.03738	0.08621	0.04348	0.08000	0.04082	0.04412	0.06250	0.06667	0.05533
S8	0.5000	0.3333	0.5000	0.5000	0.3333	1.0000	1.0000	1.0000	0.55080	0.05607	0.08621	0.04348	0.04000	0.04082	0.08824	0.06250	0.06667	0.05914
Total	8.9167	3.8667	11.5000	12.5000	8.1667	11.3333	16.0000	15.0000	8.92251	1.0000	1.0000	1.0000	1.0000	1.0000	1.0000	1.0000	1.0000	1.000000

CR = 0.0935 < 0.1																		

	**W1**	**W2**	**W3**	**W4**	**W5**	**W6**	**W7**	**λmax**	**W1**	**W2**	**W3**	**W4**	**W5**	**W6**	**W7**	**Row averages**		
W1	1.0000	1.0000	3.0000	3.0000	0.5000	2.0000	7.0000	1.5837	0.1883	0.1439	0.2432	0.2645	0.1124	0.3457	0.4504	0.2498		
W2	1.0000	1.0000	2.0000	5.0000	0.5000	0.5000	4.0000	1.3369	0.1883	0.1439	0.1622	0.4408	0.1124	0.0864	0.2574	0.1988		
W3	0.3333	0.5000	1.0000	1.0000	0.2500	1.0000	3.0000	0.6438	0.0628	0.0719	0.0811	0.0882	0.0562	0.1728	0.1930	0.1037		
W4	0.3333	0.2000	1.0000	1.0000	1.0000	0.1429	0.2000	0.5388	0.0628	0.0288	0.0811	0.0882	0.2247	0.0247	0.0129	0.0747		
W5	2.0000	2.0000	4.0000	1.0000	1.0000	1.0000	0.2000	1.7321	0.3767	0.2878	0.3243	0.0882	0.2247	0.1728	0.0129	0.2125		
W6	0.5000	2.0000	1.0000	0.1429	1.0000	1.0000	0.1429	0.9803	0.0942	0.2878	0.0811	0.0126	0.2247	0.1728	0.0092	0.1261		
W7	0.1429	0.2500	0.3333	0.2000	0.2000	0.1429	1.0000	0.2299	0.0269	0.0360	0.0270	0.0176	0.0449	0.0247	0.0643	0.0345		
Total	5.3095	6.9500	12.3333	11.3429	4.4500	5.7857	15.5429	7.0454	1.0000	1.0000	1.0000	1.0000	1.0000	1.0000	1.0000	1.0000		

CR = 0.0057 < 0.1																		

	**O1**	**O2**	**O3**	**O4**	**O5**	**O6**	**λmax**	**O1**	**O2**	**O3**	**O4**	**O5**	**O6**	**Row averages**				
O1	1.0000	5.0000	4.0000	3.0000	3.0000	3.0000	2.4638	0.4082	0.3125	0.3077	0.3448	0.3830	0.4865	0.3738				
O2	0.2000	1.0000	1.0000	0.2000	1.0000	0.3333	0.4573	0.0816	0.0625	0.0769	0.0230	0.1277	0.0541	0.0710				
O3	0.2500	1.0000	1.0000	0.5000	0.5000	0.3333	0.4466	0.1020	0.0625	0.0769	0.0575	0.0638	0.0541	0.0695				
O4	0.3333	5.0000	2.0000	1.0000	0.3333	1.0000	1.0035	0.1361	0.3125	0.1538	0.1149	0.0426	0.1622	0.1537				
O5	0.3333	1.0000	2.0000	3.0000	1.0000	0.5000	1.0371	0.1361	0.0625	0.1538	0.3448	0.1277	0.0811	0.1510				
O6	0.3333	3.0000	3.0000	1.0000	2.0000	1.0000	1.1827	0.1361	0.1875	0.2308	0.1149	0.2553	0.1622	0.1811				
Total	2.4500	16.0000	13.0000	8.7000	7.8333	6.1667	6.5909	1.0000	1.0000	1.0000	1.0000	1.0000	1.0000	1.0000				

CR = 0.0953 < 0.1																		

	T1	T2	T3	T4	T5	T6	T7	λmax	T1	T2	T3	T4	T5	T6	T7	Row averages

T1	1.0000	0.3333	0.3333	0.2000	0.3333	0.1429	0.2000	0.28396	0.0370	0.0339	0.0526	0.0278	0.0541	0.0187	0.0331	0.0367
T2	3.0000	1.0000	2.0000	1.0000	0.5000	0.5000	0.3333	0.95301	0.1111	0.1017	0.3158	0.1389	0.0811	0.0654	0.0552	0.1242
T3	3.0000	0.5000	1.0000	2.0000	1.0000	1.0000	2.0000	1.33890	0.1111	0.0508	0.1579	0.2778	0.1622	0.1308	0.3315	0.1746
T4	5.0000	1.0000	0.5000	1.0000	1.0000	1.0000	1.0000	1.05969	0.1852	0.1017	0.0789	0.1389	0.1622	0.1308	0.1657	0.1376
T5	3.0000	2.0000	1.0000	1.0000	1.0000	3.0000	0.5000	1.41990	0.1111	0.2034	0.1579	0.1389	0.1622	0.3925	0.0829	0.1784
T6	7.0000	2.0000	1.0000	1.0000	0.3333	1.0000	1.0000	1.22573	0.2593	0.2034	0.1579	0.1389	0.0541	0.1308	0.1657	0.1586
T7	5.0000	3.0000	0.5000	1.0000	2.0000	1.0000	1.0000	1.48645	0.1852	0.3051	0.0789	0.1389	0.3243	0.1308	0.1657	0.1899
Total	27.0000	9.8333	6.3333	7.2000	6.1667	7.6429	6.0333	7.76768	1.0000	1.0000	1.0000	1.0000	1.0000	1.0000	1.0000	1.0000

CR = 0.0969 < 0.1																

When examining the factors related to the weak aspects, it was found that the weakest factor with a weight of 0.2498 is “The lack of knowledge of the locals and the lack of entrepreneurship (W1)” because they cannot evaluate tourism economically and are not aware of the existing potential. Accordingly, “The fact that the carrying capacity for tourism on the island has not been determined (W5)” by 0.2125 weight, “lack of promotion and marketing activities in the region (W2)” 01988, “Lack of coordination and communication among stakeholders. “Locals, non-governmental organizations, local government and public (W6)” 0.1261 weighted as the weakest factors.

The most important opportunities for the development of rural tourism on the island are again “Increasing the interest of local tourists to the region (O1)” with 0.3738 weight and “Creating accommodation opportunities suitable for the natural and cultural texture of the region (O6)” with 0.1811 weight. These sub-factors are followed by “potential to host festivals, summer schools, workshops and various events (O4)” and “Poor transport links that preserve the island fabric (O5)” with a very low weight of 0.1537 and 0.1510, respectively.

The greatest threat was determined as “Insufficient investment in the island (T7)” with a weight value of 0.1899. The following threats are listed as “The disappearance of unprotected examples of civil architecture on the island (T5)” with a weight of 0.1784, “Losing the original cultural and social values of the villages (T3)” with a weight of 0.1746, “Young population leaving the island (T6)” with a weight of 0.1586, and “Political and economic instability on the island (T4)” with a weight of 0.1376.

After the A’WOT analysis, strategy recommendations for the sustainable development of the rural tourism destination were presented using the TOWS matrix. Although the study is examined specifically for Gökçeada, it also provides recommendations that suggest long-term and healthy development for rural tourism destinations developing in different regions.

When the TOWS matrix shown in Table 5 is examined, the strategies were determined as follows:

**TABLE 5:** TOWS Matrix for “sustainable rural tourism strategy”—produced for Gökçeada from Weihrich (1982).

Tows matrix	Strengths	Weaknesses
	S2- Natural beauty (Desire to be within natural life)	W1- Lack of knowledge and entrepreneurship of the locals
	S1- The effectiveness of traditional architecture	W5- The fact that the carrying capacity for tourism on the island has not been determined
	S6- Popular destination	W2- Lack of promotion and marketing activities in the region
	S5- The continuation of intercultural interaction on the island	W6- Lack of coordination and communication among stakeholders. (local people, non-governmental organizations, local government and public)

**Opportunities**	**SO strategy**	**WO strategy**

		
O1- Increasing the interest of local tourists to the region	“Creating a sustainable tourism management with low impact on the environment”	“Improving the touristic infrastructure and determining the capacity”
O6-Creating accommodation opportunities suitable for the natural and cultural texture of the region	Strategy 1	Strategy 2
O4- Potential to host festivals, summer schools, workshops and various events	(S2,S1,S6,O1,O6,O4,O5)	(O1,O6,O4, W1,W5,W2)
O5- Poor transport links that preserve the island fabric	To create an infrastructure to develop alternative tourism types. Preferring architectural designs suitable for the local texture. To evaluate the existing building stock as accommodation facilities.	More investments should be made in the development of tourism infrastructure and preserving the natural, cultural and historical texture of the island. National and international events promoting the island should be organized.

**Threats**	**ST strategy**	**WT strategy**

		
T7- Insufficient investment for the island	“Protection of social and cultural values”	“Developing tourism investment and improvement policies with the participation of local residents”
T5- The disappearance of unprotected examples of civil architecture on the island	Strategy 3	Strategy 4
T3- Losing the original cultural and social values of the villages	(S2,S1,S5,T5,T3)	(W1,W5,W2,W6,T7,T3,T6)
T6- The young population leaving the island		All stakeholders should take part together in activities for the development of the region. Business and entrepreneurship trainings should be organized for the island residents. The executive mechanism should be strengthened.

• SO Strategy “Building a sustainable tourist management with low impact on the environment”

Rural tourism on the island, where organic products, rural lifestyle and clean nature can be presented together, as well as cultural tourism where rich architecture, historical, and cultural diversity will be presented, and sea tourism with underwater diving, windsurfing, angling and clean beaches are the leading alternative tourism types is on the plan. The activities determined while developing all these types of tourism should be created by considering the environmental effects. Tourism should not only focus on economic benefits, but should also consider environmental and social benefits. With sustainable development, a potential environmental threat assessment system should be established that could reflect the material and energy inputs and outputs in tourism, tourist capacity and the limits of environmental degradation in a certain range and to a certain extent (Zhang, 2012).

• WO Strategy “Improving the touristic infrastructure and determining the capacity”

Alternative tourism types on the island should be diversified and promotions should be made for the island (sea tourism, religious tourism, nature tourism (ecotourism) and historical tourism etc.). Tourist routes should be determined and the daily optimal tourist capacity and maximum tourist capacity should be evaluated at a touristic point. Efforts should be made to preserve the cultural and historical texture of the island. Policies should be established to protect the nature and resources of the island and to establish facilities in a way that does not hinder the economic and social development of the island. It will be an important strategy to make investments in cultural activities by spreading the festivals to all seasons and to involve tourists in rural life with various activities.

• ST Strategy “Protection of social and cultural values”

The Greek culture, which was dominant on the island, was blended with the cultures brought by the Black Sea, Eastern Anatolia and Bulgarian immigrants who were later settled on the island, and a new cultural texture unique to the island was formed. This cultural richness and diversity of the island should be considered as a whole with its natural, socio-cultural, administrative and architectural aspects and strategies for protection should be developed. Residences, churches, chapels, mosques, shops, coffee houses, mills, olive oil and soap factories and laundries, which are the achievements of multiculturalism, should be protected and brought into tourism.

• WT Strategy “Developing tourism investment and improvement policies with the participation of local residents”

Suggestions should be developed to transform tourism activities into an economic contribution for the local people, and the living standards of the local people should be increased. In the development of tourism on the island, the involvement of all stakeholders who are affected or affected by tourism and their inclusion in the decision processes will ensure a sustainable development of tourism. This will encourage local people and other community organizations to take ownership of the island’s tourism resources.

## Conclusion

The health and economic problems that came with COVID-19 have brought alternatives to tourism activities on the agenda again. Movement restrictions between countries have increased rural tourism activities at the local level, especially in developing countries. Making this mobility in domestic tourism permanent and using the potentials in rural areas correctly with the increase in infrastructure, organization and knowledge will turn the crisis experienced in this period into an opportunity. In the case study conducted in Turkey, they stated that tourists feel safer in unexplored coastal areas with less human density than in heavily used coastal areas, and in addition, they experience many new historical, cultural, natural and gastronomic discoveries. For this reason, Gökçeada, with its unspoiled nature, has started to attract the attention of those living in crowded cities, especially during the pandemic process. However, sustainable and credible strategies must be established on the island to seize new opportunities created by the devastating and changing impact of the pandemic. With the study carried out in Gökçeada, the following strategies are recommended for sustainable development for rural tourism.

• A more conscious and systematic development will be achieved by making the right investments in the island. Increasing employment opportunities will prevent the young population from leaving the island.
• Access to the island is limited and inconvenient. Although this situation may seem negative at first glance, it should be turned into an important opportunity for the island by contributing to the island becoming a center of attraction for people who want to be closer to nature and have adopted a healthy, peaceful and humble lifestyle.
• It is possible to minimize the damage to nature with the right decisions to be taken at the design stage in order to preserve the ecological identity of this newly developing island.
• The architectural textures of the traditional Gökçeada houses are the unique local values. Reflecting this texture in new buildings and preserving old buildings will make an important contribution to the sustainable development of the island by keeping the local structure and life identity alive. Many traditional stone houses in Gökçeada remain idle. It is very important for the protection of the ecosystem to use the existing houses by repairing instead of opening new settlements. For this reason, the restoration of these houses and their use as second houses, boutique hotels, hostels, cafeterias and bringing them to tourism will both keep the existing architecture alive and contribute to the development of tourism without disturbing the natural balance.
• The fact that the island has many alternative tourism types increases the interest in the region and it is seen positively in terms of spreading tourism to all seasons. The potential to host festivals, summer schools, workshops and various events increases the interest of tourists.
• The continuation of intercultural interaction on the island increases the diversity and cultural richness. Greek villages have a dominant place in Gökçeada culture. In particular, the fact that the first settled people were of Greek origin, and the Christian-Orthodox sects were represented on the island at the metropolitan level, which contributed to the increase in the importance given to religious ceremonies. Fairs held after religious ceremonies increase cultural fusion in the island.
• Continuous and balanced development as well as economic development guided by ecological principles should be adopted in the new planning of the island.

It is hoped that this study, which is carried out in Turkey, will be an example for new studies to be made for rural tourism destinations. During the Pandemic, international restrictions have occurred all over the world, and these restrictions will continue in case of new global epidemics. In order to turn this negative situation into an opportunity, a holistic perspective is needed for the sustainable development of tourism. Tourism development increases business sales revenues, stimulates local production, creates new job and investment opportunities, and increases government revenues through taxation. However, in addition to these benefits, uncontrolled development can also cause social and environmental problems. In order to minimize these problems, it is necessary for governments to create a number of new policies and provide financial support. The literature research and field study reveal that the problems identified for rural tourism, especially in developing countries, are similar. Therefore, it is thought that it will be beneficial to consider the following recommendations in the development of sustainable rural tourism strategies.

• First of all, rural accommodation opportunities should be increased and rural tourism should be marketed effectively, especially in developing countries.
• Most of those working in the tourism sector work part-time. Continuing tourism activities throughout the year and training employees in this sector is an important strategy in ensuring sustainability.
• Local people should be given active tasks by taking into account traditional culture and values in the studies for the development of rural tourism. All stakeholders should act together in developing infrastructure, institutional framework, marketing and cooperation.
• The rural environment is fragile and vulnerable to potential damages that may result from the development of tourism. The natural environment may suffer while meeting the needs of large numbers of tourists. For this reason, tourist carrying capacities must be determined in advance and all planning should be made according to this capacity when opening rural areas to tourism.
• Conservation and development of natural resources should be recognized as an important component in the dynamics of the tourism industry.
• The inexperience of the local people has pushed the people of the region out of tourism earnings in rural tourism management. Raising awareness of the local people and focusing on the local workforce is an important strategy for the sustainability of rural tourism.

## Data availability statement

The raw data supporting the conclusions of this article will be made available by the authors, without undue reservation.

## Ethics statement

Ethical review and approval was not required for the study on human participants in accordance with the local legislation and institutional requirements. Written informed consent from the participants was not required to participate in this study in accordance with the national legislation and the institutional requirements.

## Author contributions

FK, FC, MD-R, and JÁ-G: conceptualization, investigation, methodology, formal analysis, writing—original draft, preparation and review and editing. All authors have read and agreed to the published version of the manuscript.
